# Supplementary figures and images for: Peroxisomal import is circadian in glia and regulates sleep and lipid metabolism
Source: PLoS Biol. 2026 Jul 15;24(7):e3003901. doi: 10.1371/journal.pbio.3003901 (PMC13387613; doi:10.1371/journal.pbio.3003901)

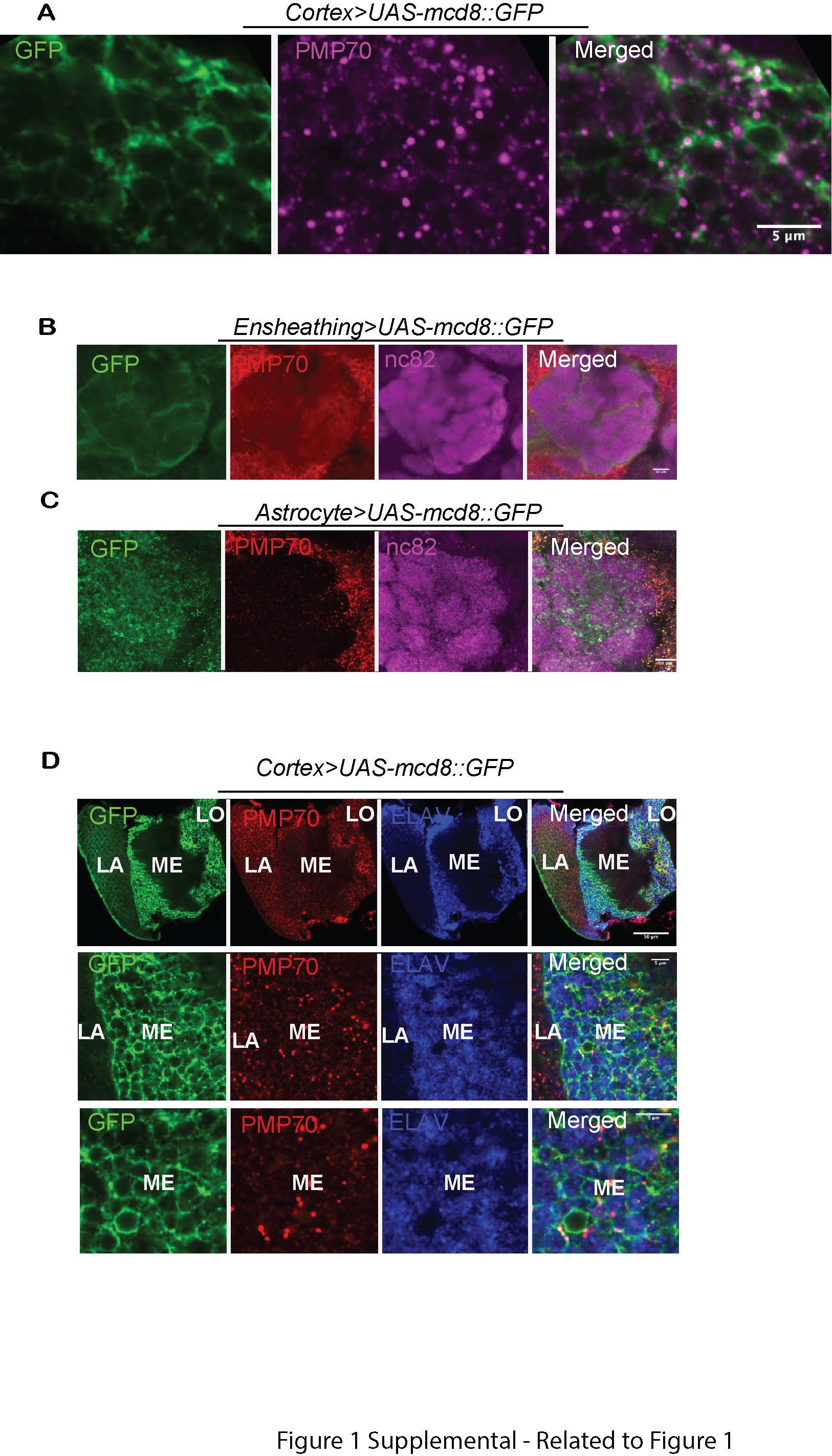

Supplement: S1 Fig — Representative images comparing central brain glial classes, with colocalization between glial driven GFP(green) counterstained with PMP70(magenta). PMP70 peroxisomes are abundant inside cortex glial regions as compared to astrocyte and ensheathing glia. nC82(magenta) marks the pre-synaptic active zones representing the neuropils of the adult Drosophila brain. (A) Adult female Drosophila brains (7 days old) Cortex glia (GMR54H02-GAL4 > UAS- mCD8::GFP) tracking colocalization between GFP(green) counterstained with PMP70(magenta). (B) Adult female Drosophila brains (7 days old) Ensheathing glia (GMR56F03-GAL4 > UAS-mCD8::GFP) tracking colocalization between GFP(green) counterstained with PMP70(red) and nc82 (magenta). (C) Adult female Drosophila brains (7 days old) astrocytic glia (alrm-GAL4 > UAS- mCD8::GFP) tracking colocalization between GFP(green) counterstained with PMP70(red) and nc82 (magenta). (D) Adult female Drosophila brains (7 days old) Cortex glia (GMR54H02-GAL4 > UAS- mCD8::GFP) representative image showing the optic lobe regions (LA-lamina; ME- medulla; LO-lobula) showing PMP70 (red) positive puncta in GFP(green) rich regions of cortex. (TIF) [file pbio.3003901.s003.tif]

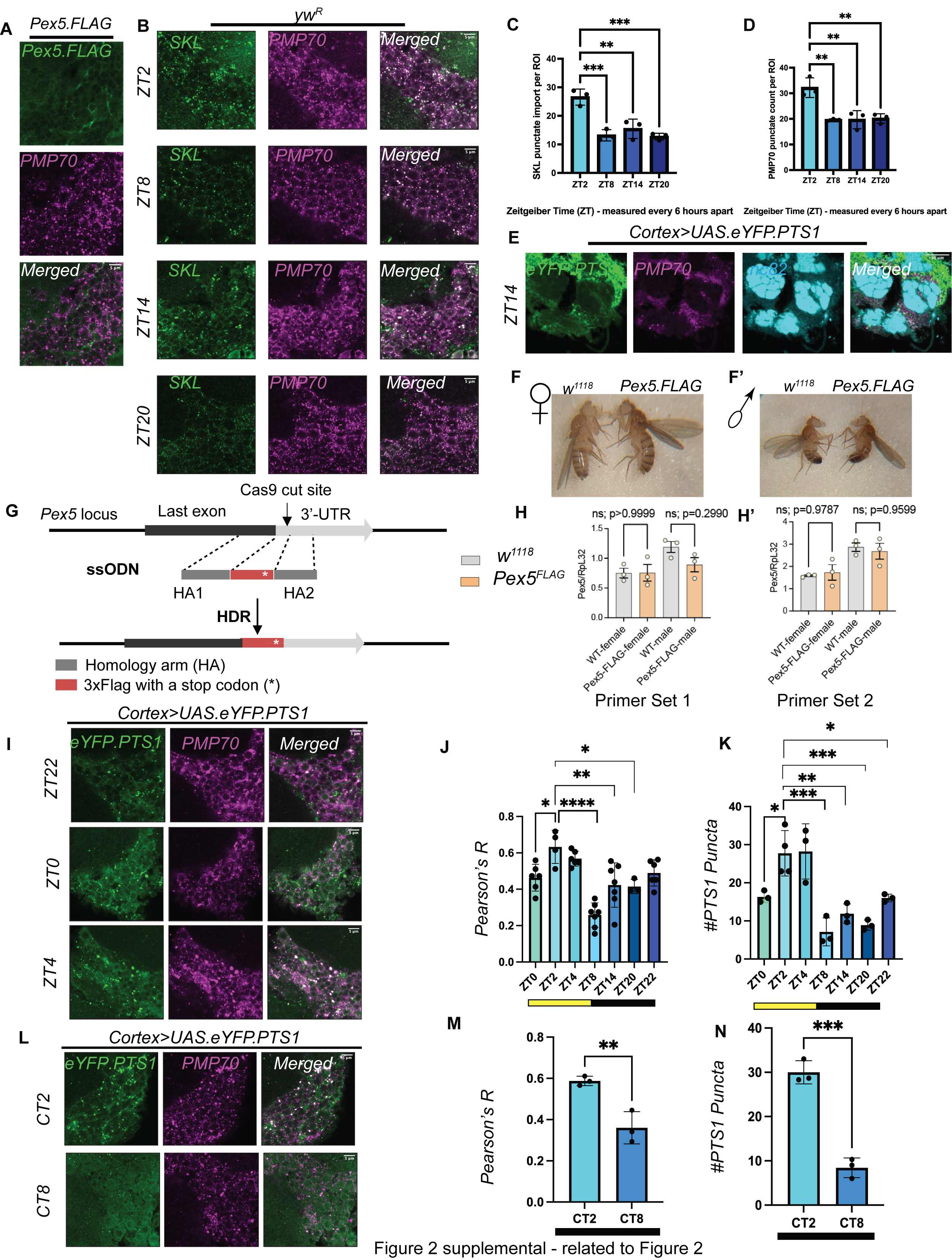

Supplement: S2 Fig — (A) Adult female Drosophila brains (7 days old) tracking Pex5.FLAG knock-in flies co- stained with anti-FLAG(green) and anti-PMP70(magenta). (B) Adult female Drosophila brains (7 days old) ywR wildtype flies dissected across ZT2, ZT8, ZT14 and ZT20 and stained with anti-SKL(green), anti-PMP70(magenta) to show circadian oscillations of peroxisomal import peak at ZT2 and trough at ZT8. (C) Quantification in ywR wildtype flies of SKL puncta at ZT2, ZT8, ZT14, ZT20. n ≥ 3 brains per genotype, dots represent biological replicates averaged over ≥ 3 ROIs. ROIs were taken from zooms into cortex below the antennal lobes and above the suboesophageal ganglion. (D) Quantification in ywR wildtype flies of PMP70 puncta at ZT2, ZT8, ZT14, ZT20; n ≥ 3 brains per genotype, dots represent biological replicates averaged over ≥3 ROIs. (E) Adult female Drosophila brains (7 days old) tracking peroxisomal import oscillation at ZT14 observed in glia with UAS-eYFP.PTS1 using GMR54H02-GAL4. eYFP-tagged PTS1-protein import (green) is counterstained with PMP70 (magenta), an abundant peroxisomal membrane protein to detect peroxisomes along with anti-nc82. Scale bar = 50μm. (F) Adult female Drosophila comparing w1118 versus Pex5.FLAG. (F′) Adult male Drosophila comparing w1118 versus Pex5.FLAG. (G) CRISPR/Cas9-based knock-in strategy to tag Pex5 with a 3XFLAG epitope. (H) Relative transcript expression of Pex5 (Primer set 1) in w1118 versus Pex5.FLAG measured by RT-qPCR (full flies, both adult female and male; 7 days old) at ZT2. (H′) Relative transcript expression of Pex5 (Primer set 2) in w1118 versus Pex5.FLAG measured by RT-qPCR (full flies, both adult female and male; 7 days old) at ZT2. (I) Adult female Drosophila brains (7 days old) Cortex>UAS-eYFP.PTS1 flies dissected across ZT22, ZT0 and ZT4 stained with anti-GFP(green), anti-PMP70(magenta). (J) Quantification of eYFP-PTS1 and PMP70 Pearson colocalization. n ≥ 3 brains per genotype, data points represent biological replicates. (K) Quant [file pbio.3003901.s004.tif]

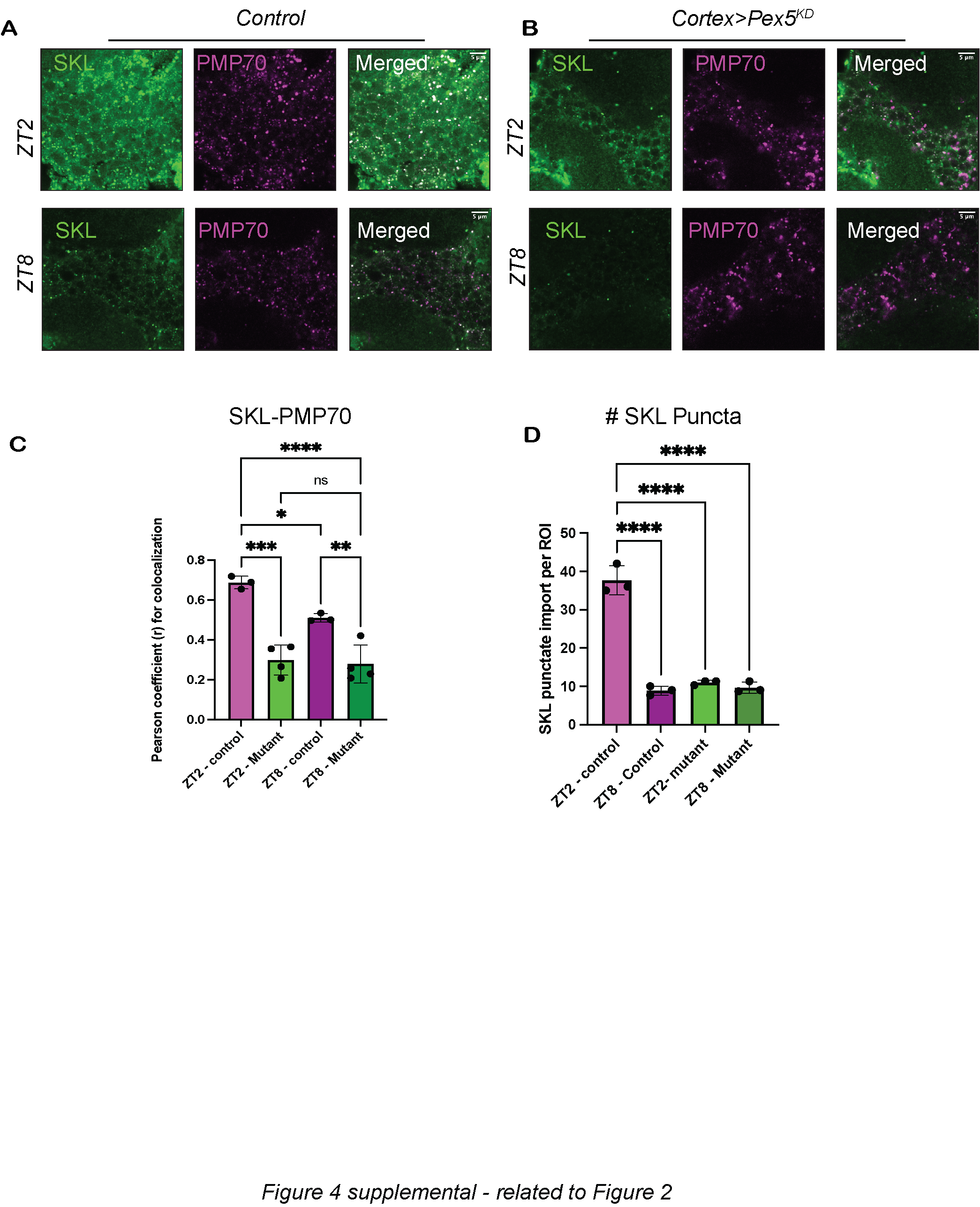

Supplement: S4 Fig — (A) Adult female Drosophila brains (7 days old) Control (w1118/UAS-Pex5 RNAi) flies dissected across ZT2 and ZT8 stained with anti-SKL(green), anti-PMP70(magenta) to show circadian oscillations of peroxisomal import peak at ZT2 and trough at ZT8. (B) Adult female Drosophila brains (7 days old) mutants (Cortex > UAS-Pex5 RNAi; (BDSC# 55322) flies dissected across ZT2 and ZT8 stained with anti-SKL(green), anti-PMP70(magenta). (C) Quantification of SKL and PMP70 Pearson colocalization. n ≥ 3 brains per genotype, data points represent biological replicates. ROIs were taken from zooms into cortex below the antennal lobes and above the suboesophageal ganglion, and quantified from 200 × 200 pixel windows inside these zooms. (D) Quantification of the number of SKL punctate. n ≥ 3 brains per genotype, dots represent biological replicates averaged over ≥3 ROIs. * p < 0.05, ** p < 0.01, *** p < 0.001, **** p < 0.0001 by Tukey’s multiple comparisons test. The data underlying this figure can be found in S1 Data. (TIF) [file pbio.3003901.s006.tif]

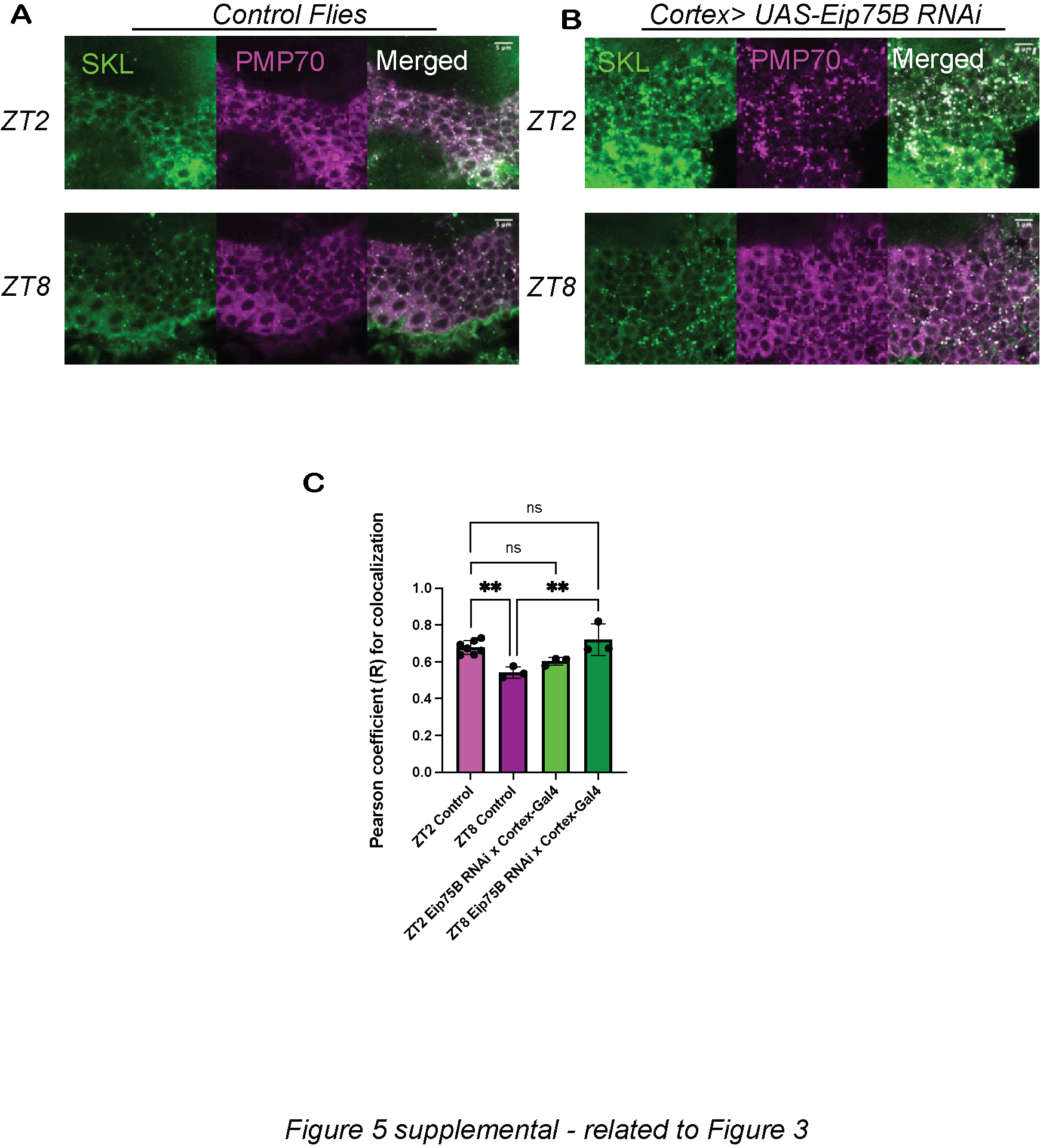

Supplement: S5 Fig — (A) Adult female Drosophila brains (7 days old) dissected at ZT2 and ZT8 for control flies (w1118/UAS-Eip75B RNAi) stained with anti-SKL(green) and anti-PMP70(magenta). (B) Adult female Drosophila brains (7 days old) dissected at ZT2 and ZT8 for GMR54H02- Gal4 > UAS-Eip75B RNAi knockdown flies. (C) Quantification Pearson R coefficient colocalization calculated (see methods) for GMR54H02-Gal4 cross with UAS-Eip75B RNAi knockdown flies compared with control flies at ZT2, ZT8; n ≥ 3 brains per genotype, dots represent biological replicates averaged over ≥3 ROIs. ROIs were taken from zooms into cortex below the antennal lobes and above the suboesophageal ganglion.* p < 0.05, ** p < 0.01, *** p < 0.001, **** p < 0.0001 by Tukey’s multiple comparisons test. The data underlying this figure can be found in S1 Data. (TIF) [file pbio.3003901.s007.tif]

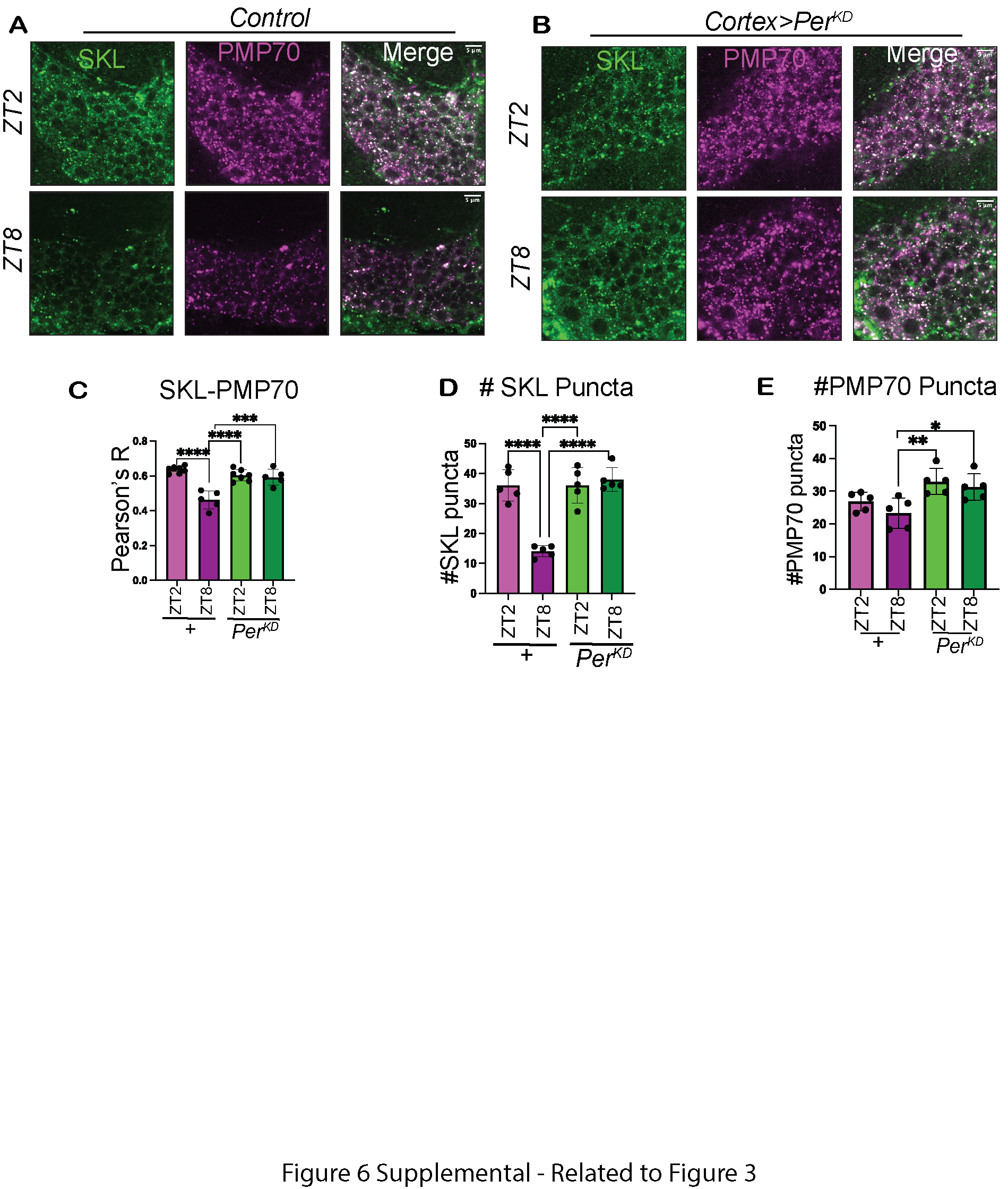

Supplement: S6 Fig — Per RNAi autonomous control in cortex glia. (A) Adult female Drosophila brains (7 days old) tracking peroxisomal import oscillation across ZT2, ZT8 in Control >w1118/GMR54H02-Gal4) with SKL (green) counterstained with PMP70 (magenta). (B) Adult female Drosophila brains (7 days old) tracking peroxisomal import oscillation across ZT2, ZT8 in Cortex-Glia> UAS-Per RNAi (GMR54H02-Gal4 > UAS-Per RNAi) with SKL (green) counterstained with PMP70 (magenta). (C) Quantification of SKL and PMP70 Pearson colocalization. n ≥ 3 brains per genotype, data points represent biological replicates averaged over ≥3 ROIs. ROIs were taken from zooms into cortex below the antennal lobes and above the suboesophageal ganglion, and quantified from 200 × 200 pixel windows inside these zooms. (D) Quantification of the number of SKL punctate. n ≥ 3 brains per genotype, dots represent biological replicates averaged over ≥3 ROIs. (E) Quantification of the number of PMP70 punctate. n ≥ 3 brains per genotype, dots represent biological replicates averaged over ≥3 ROIs. * p < 0.05, ** p < 0.01, *** p < 0.001, **** p < 0.0001 by Tukey’s multiple comparisons test. The data underlying this figure can be found in S1 Data. (TIF) [file pbio.3003901.s008.tif]

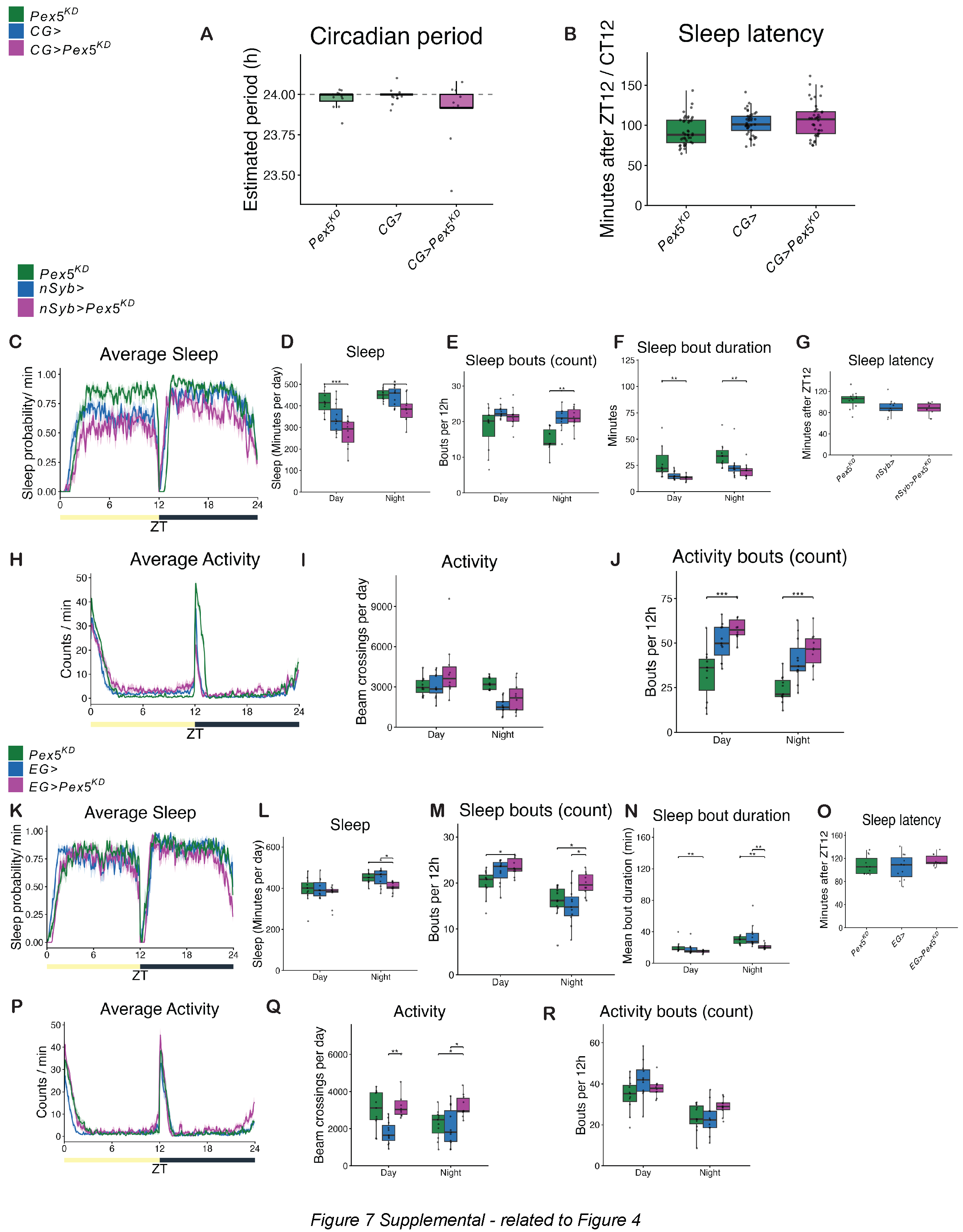

Supplement: S7 Fig — Loss of Pex5 sleep and activity analyzed for neuronal and ensheathing glia. (A) Circadian period length measured in 12-hour/12-hour, dark/dark cycle on an average for 5–6 days continuously for 5-day to 10-day old males post-eclosion after initial entrainment in 12-hour/12-hour, light/dark for cortex glia driver GMR77A03-GAL4 crossed with UAS-Pex5 RNAi KD (magenta) compared to parental controls UAS-Pex5-RNAi (green) and GMR77A03-GAL4 (blue). (B) Sleep latency was measured after lights off in 12-hour/12-hour, light/dark cycle on an average for 4–5 days continuously for 5-day to 10-day old males post-eclosion for cortex glia driver GMR77A03-GAL4 crossed with UAS-Pex5 RNAi KD (magenta) compared to parental controls UAS-Pex5-RNAi (green) and GMR77A03-GAL4 (blue). (C) Average Sleep (sleep probability/min) measured in 12-hour/12-hour, light/dark cycle on an average for 4–5 days continuously for 5-day to 10-day old males post-eclosion. Average sleep time-plot showing sleep probability/min (bouts of inactivity > 5 min) for the neuronal driver nSyb-GAL4 crossed with UAS-Pex5 RNAi KD (magenta) compared to parental controls UAS-Pex5-RNAi (green) and nSyb-GAL4 (blue). (D) Total sleep(minutes per day) quantified for all three genotypes from (C). Individual black dots represent different biological replicates. (E) Sleep bouts per 12hour quantified for all three genotypes from (C). Individual black dots represent different biological replicates. (F) Sleep bout duration per minute quantified for all three genotypes from (C). Individual black dots represent different biological replicates. (G) Sleep latency quantified for all three genotypes from (C). Individual black dots represent different biological replicates. (H) Average activity measured in 12-hour/12-hour, light/dark cycle on an average for 4–5 days continuously for 5-day to 10-day old males post-eclosion. Average activity time-plot showing counts/min (bouts of inactivity > 5 min) for the neuronal driver nSyb-GAL4 crossed wit [file pbio.3003901.s009.tif]

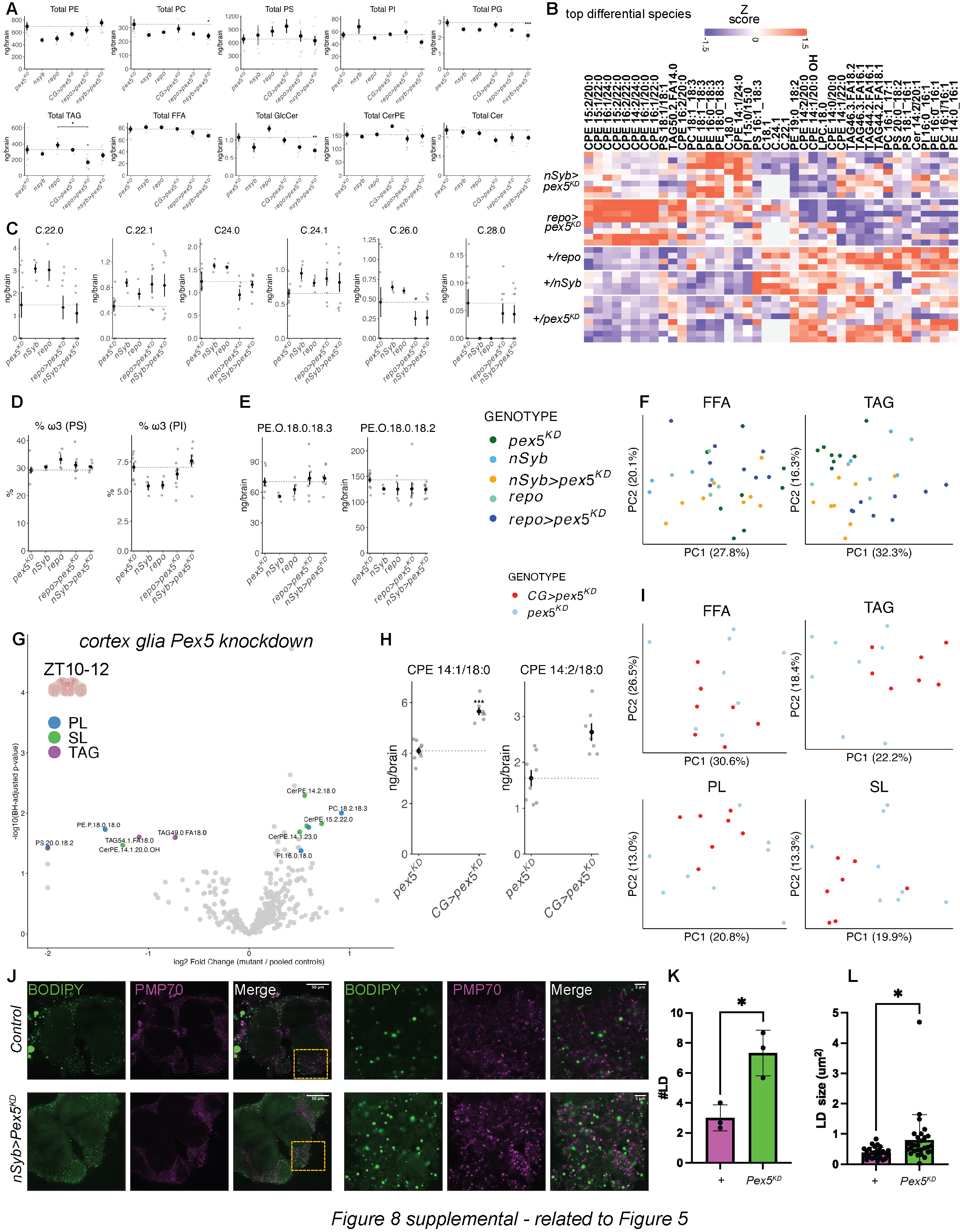

Supplement: S8 Fig — Pex5 is essential for lipid homeostasis in the adult brain. (A) Total ng/brain for lipid families analyzed by LC–MS/MS of day 7 female brains at ZT10–12; see S1 Table for full genotypic details. n ≥ 4 tubes of 20 brains for each genotype. (B) Heatmap of top 30 species changed between controls and mutants, z-scored by relative % abundance. (C) Free fatty acid analysis of longer chain fatty acid species across genotype, including very long chain fatty acids (VLCFA) (ng/brain). (D) % of ω3 fatty acids in phosphatidylserine (PS) and phosphatidylinositol (PI). (E) Major alky-ether phospholipids shown in ng/brain across genotypes. (F) Principal component analysis of TAGs and free fatty acids (FFA) colored by genotype. (G) Volcano plot showing p-value versus log2 fold change for cortex glia pex5 knockdown versus parental controls. Phospholipids are colored green, sphingolipids colored green, and TAG colored purple. (H) Differentially abundant ceramide phosphoethanolamine (CPE) species in CG > pex5KD brains. (I) principal component analysis of controls versus CG > pex5KD brains (red) versus control (grey) for sphingolipids (SL), phospholipids (PL), TAG, and free fatty acids (FFA). (J) Adult female Drosophila brains (7 days old) tracking neutral lipid droplet (BODIPY 493/503, green) counterstained with PMP70 (magenta). Representative images of UAS- control (UAS-Pex5 RNAi > w1,118) versus neuronal specific knockdown of Pex5 using nSyb-Gal4 > UAS-Pex5 RNAi. (K) Quantification of different lipid droplet size(um2) from J. n ≥ 3 brains per genotype, dots represent individual lipid droplets per biological replicates averaged over ≥3 ROIs. ROIs were taken from zooms into cortex below the antennal lobes and above the suboesophageal ganglion, and quantified from 200 × 200 pixel windows inside these zooms. (L) Quantification of lipid droplet count from J. n ≥ 3 brains per genotype, dots represent number of lipid droplets per biological replicates averaged over ≥3 ROIs. * p < 0.05, ** [file pbio.3003901.s010.tif]
