# Supplementary material for: Peroxisomal import is circadian in glia and regulates sleep and lipid metabolism
Source: PLoS Biol. 2026 Jul 15;24(7):e3003901. doi: 10.1371/journal.pbio.3003901 (PMC13387613; doi:10.1371/journal.pbio.3003901)
Supplement: S3 Fig — (A) Global heatmap of peroxisomal gene expression extracted from fly-sleep single across all glial clusters to show Pex5 expression is highest in CXG (cortex-glia) cluster. (B) Global heatmap of peroxisomal gene expression extracted from fly-sleep single across neuronal clusters to show low Pex5 expression as opposed to CXG glial cluster. (PDF) [file pbio.3003901.s005.pdf]

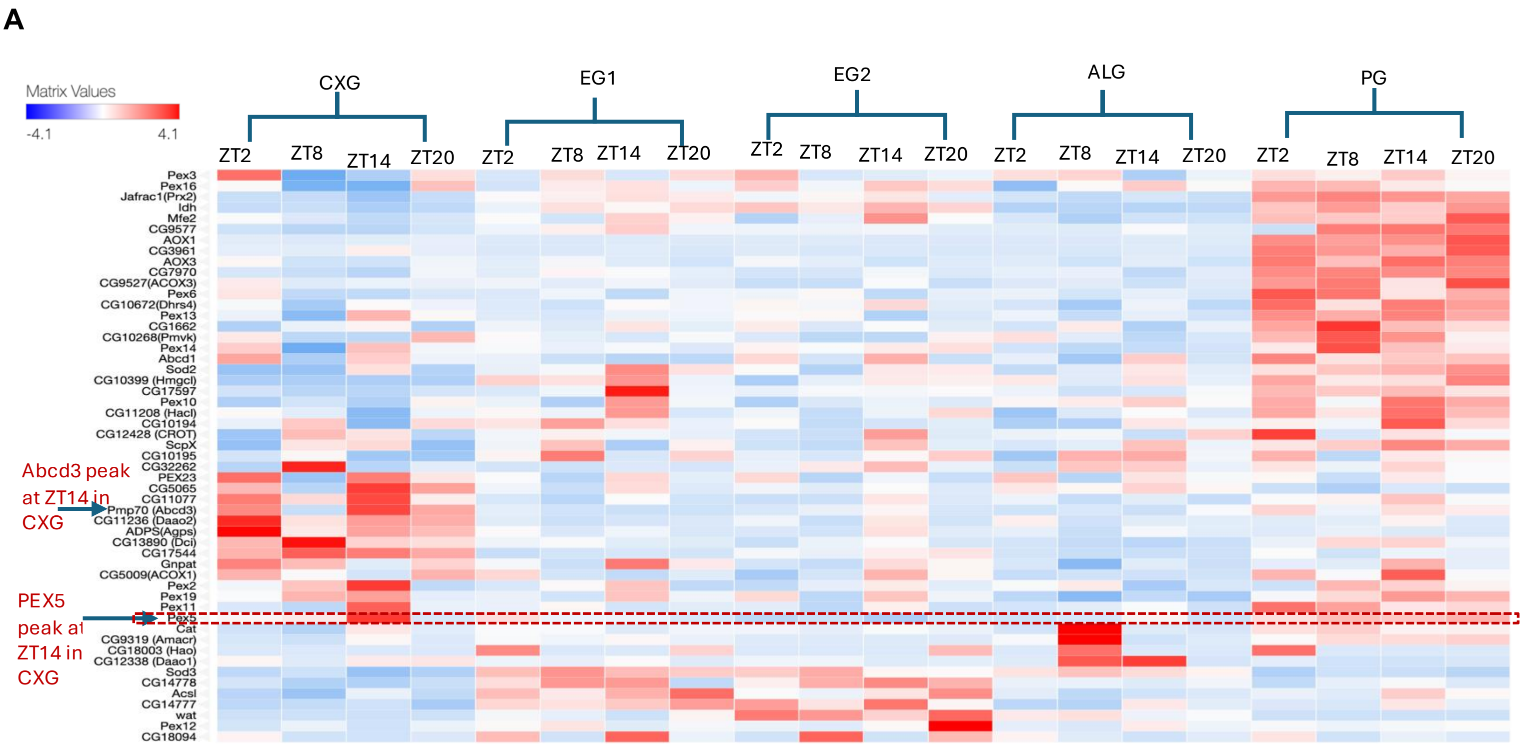

Global Heat map of peroxisomal differentially expressed genes in glial clusters at different ZT (zeitgeber) time points from Drosophila sleep single-cell

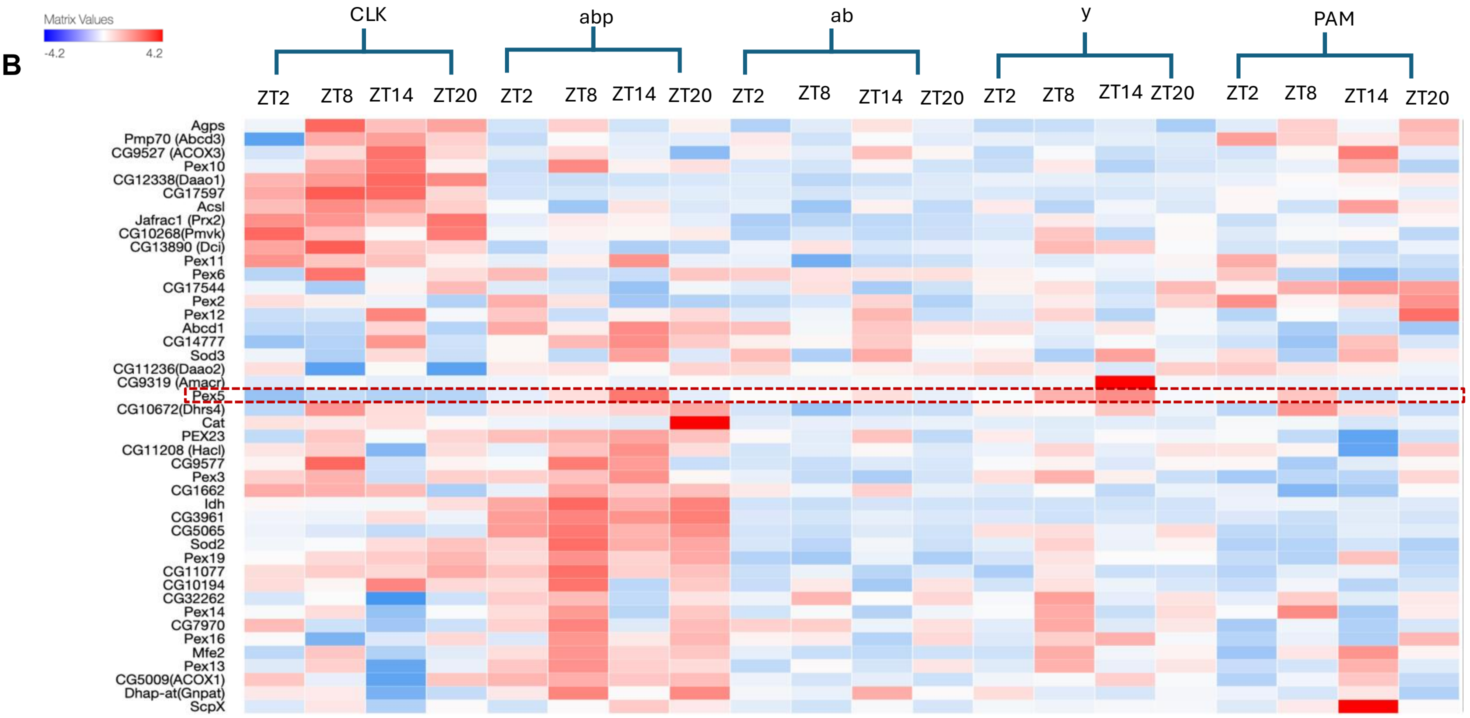

Global Heat map of peroxisomal differentially expressed genes in Neuronal clusters from Drosophila sleep single-cell

Figure 3 Supplemental - related to figure 2
